# Genome Sequence and Annotation of *Acremonium chrysogenum*, Producer of the β-Lactam Antibiotic Cephalosporin C

**DOI:** 10.1128/genomeA.00948-14

**Published:** 2014-09-25

**Authors:** Dominik Terfehr, Tim A. Dahlmann, Thomas Specht, Ivo Zadra, Hubert Kürnsteiner, Ulrich Kück

**Affiliations:** a Christian Doppler Laboratory for “Fungal Biotechnology,” Lehrstuhl für Allgemeine und Molekulare Botanik, Ruhr-Universität Bochum, Bochum, Germany; b Sandoz GmbH, Kundl, Austria

## Abstract

The filamentous fungus *Acremonium chrysogenum* is the industrial producer of the β-lactam antibiotic cephalosporin C. Here, we present the genome sequence of strain ATCC 11550, which contains genes for 8,901 proteins, 127 tRNAs, and 22 rRNAs. Genome annotation led to the prediction of 42 gene clusters for secondary metabolites.

## GENOME ANNOUNCEMENT

*Acremonium chrysogenum* is an imperfect ascomycete which was first described in 1954 by Guiseppe Brotzu who isolated this fungus from Sardinian coastal seawater. Later the antibiotic potential of its extracts was analyzed and described (1, 2). In contrast to the commonly used β-lactam antibiotic penicillin, cephalosporin C is active against both Gram-negative and Gram-positive bacteria. The ongoing demand for antibiotic chemotherapy has generated a world market where β-lactam antibiotics are the most commonly used drugs with an estimated annual turnover of US$ 22 billion and an estimated market share of 50% by cephalosporin C derivatives (3).

Previous studies on the karyotype from the ATCC 11550 strain reveal an estimated genome size of 32.7 Mb on 8 chromosomes (4) and subsequent progress in developing molecular tools allowed the genetic manipulation of this industrial fungus (5).

In this study, we sequenced the genome of the *A. chrysogenum* ATCC 11550 strain in a whole-genome shotgun sequencing approach which delivered ~23.4 million paired reads with a median insert size of 266 bp and ~12.6 million paired reads with a median insert size of 7,704 bp. The acquired sequence reads were assembled into 2,799 contigs using Velvet v1.2.10 with a k-mer length of 63 nt (6). These were subsequently assembled into 541 scaffolds using SSPACE v2.0 (7). The resulting genome sequence has an estimated size of 28.6 Mb (*N*_50_ 166,906 bp, *N*_Max_ 878,651 bp, mean coverage 137.1) with 1,189 gaps and a G+C content of 54.6%. Within these sequences scaffold 543 contains the complete mitochondrial genome.

The protein coding genes were predicted using the MAKER annotation pipeline v2.31.5 in collaboration with Augustus v2.5.5 and SNAP resulting in 8,901 protein coding sequences with minimal protein lengths of 25 amino acids (8–10). A total of 5,433 (61%) from these putative protein coding genes were successfully annotated via BLASTp similarity searches against the Swiss-Prot database. Furthermore, 127 tRNA and 22 rRNA genes where predicted using tRNAscan-SE v1.3.1 and RNAmmer v1.2 (11, 12).

To evaluate the potential to produce secondary metabolites the genome sequence was also used for secondary metabolite cluster prediction with antiSMASH v2.0 (13). Overall 42 secondary metabolite clusters were predicted which were subcategorized as 14 type 1 polyketide synthetase clusters, 10 terpene synthase clusters, 7 nonribosomal peptide synthetase clusters, 8 hybrid clusters, and 3 not further specified secondary metabolite clusters.

### Nucleotide sequence accession numbers.

This whole-genome shotgun project has been deposited at DDBJ/ENA/GenBank under the accession no. JPKY00000000. The version described in this paper is the first version, JPKY01000000.
